# Concrete Crack Detection and Segregation: A Feature Fusion, Crack Isolation, and Explainable AI-Based Approach

**DOI:** 10.3390/jimaging10090215

**Published:** 2024-08-31

**Authors:** Reshma Ahmed Swarna, Muhammad Minoar Hossain, Mst. Rokeya Khatun, Mohammad Motiur Rahman, Arslan Munir

**Affiliations:** 1 Department of Computer Science and Engineering, Mawlana Bhashani Science and Technology University, Tangail 1902, Bangladesh; reshmaahmed.sw@gmail.com (R.A.S.); minoarhossain16005@gmail.com (M.M.H.); motiurcse@mbstu.ac.bd (M.M.R.); 2 Department of Computer Science and Engineering, Bangladesh University, Dhaka 1000, Bangladesh; rokeya2kcse@gmail.com; 3 Department of Electrical Engineering and Computer Science, Florida Atlantic University, Boca Raton, FL 33431, USA

**Keywords:** feature fusion, curvelet transform, convex hull, crack recognition, explainable AI, LDA

## Abstract

Scientific knowledge of image-based crack detection methods is limited in understanding their performance across diverse crack sizes, types, and environmental conditions. Builders and engineers often face difficulties with image resolution, detecting fine cracks, and differentiating between structural and non-structural issues. Enhanced algorithms and analysis techniques are needed for more accurate assessments. Hence, this research aims to generate an intelligent scheme that can recognize the presence of cracks and visualize the percentage of cracks from an image along with an explanation. The proposed method fuses features from concrete surface images through a ResNet-50 convolutional neural network (CNN) and curvelet transform handcrafted (HC) method, optimized by linear discriminant analysis (LDA), and the eXtreme gradient boosting (XGB) classifier then uses these features to recognize cracks. This study evaluates several CNN models, including VGG-16, VGG-19, Inception-V3, and ResNet-50, and various HC techniques, such as wavelet transform, counterlet transform, and curvelet transform for feature extraction. Principal component analysis (PCA) and LDA are assessed for feature optimization. For classification, XGB, random forest (RF), adaptive boosting (AdaBoost), and category boosting (CatBoost) are tested. To isolate and quantify the crack region, this research combines image thresholding, morphological operations, and contour detection with the convex hulls method and forms a novel algorithm. Two explainable AI (XAI) tools, local interpretable model-agnostic explanations (LIMEs) and gradient-weighted class activation mapping++ (Grad-CAM++) are integrated with the proposed method to enhance result clarity. This research introduces a novel feature fusion approach that enhances crack detection accuracy and interpretability. The method demonstrates superior performance by achieving 99.93% and 99.69% accuracy on two existing datasets, outperforming state-of-the-art methods. Additionally, the development of an algorithm for isolating and quantifying crack regions represents a significant advancement in image processing for structural analysis. The proposed approach provides a robust and reliable tool for real-time crack detection and assessment in concrete structures, facilitating timely maintenance and improving structural safety. By offering detailed explanations of the model’s decisions, the research addresses the critical need for transparency in AI applications, thus increasing trust and adoption in engineering practice.

## 1. Introduction

Concrete is a ubiquitous material in the construction industry, which is valued for its strength and durability. Like all materials, it is susceptible to damage over time, with cracks being a common and critical form of deterioration. Cracks can indicate underlying issues, such as structural weaknesses, material deterioration, and environmental damage, which, if left unaddressed, can lead to severe structural failures and safety hazards for occupants [1,2]. In 2013, a building named Rana Plaza in Bangladesh fell down because of cracks, causing 1134 deaths [3]. Likewise, in 2021, a building in Miami collapsed, mainly due to cracks in its foundation and support columns, resulting in 98 deaths [4]. Many other examples of similar damage exist, not just for buildings but also for dams, roads, and so on [5,6]. So, early detection and precise localization of these cracks are essential for maintaining structural integrity and ensuring safety that allows for timely repairs, preventing further deterioration and reducing repair costs [7]. Traditional methods of crack detection rely on human inspectors, whose assessments can be subjective, inconsistent, and time-consuming, especially for large structures or hard-to-reach areas [8]. To overcome these issues, recent advancements in artificial intelligence (AI) and computer vision offer promising solutions. AI-based crack detection utilizes intelligent mechanisms to analyze images of concrete surfaces, providing automated, accurate, and comprehensive assessments of cracks. These technologies enhance the precision and efficiency of crack detection, minimizing human error and subjectivity, which allows for continuous monitoring without the need for constant human intervention [9,10]. Hence, this research aims to generate an intelligent approach to detect, localize, and explain the cracks in concrete surfaces.

In the past few years, numerous intelligent approaches have emerged for detecting building cracks from images. Akgül [11] developed a fused CNN model by merging two existing architectures known as MobileNetV2 and DenseNet169 for detecting surface cracks in concrete buildings. This model achieved an overall accuracy of 99.87% with fewer features and less complexity compared to other CNN techniques. Additionally, several state-of-the-art (SOTA) CNN models were evaluated in this research. Padsumbiya et al. [12] proposed a CNN-based technique to detect surface cracks within a building from lower-resolution images. This lower-resolution image handling made their method cost-effective and less complex. Additionally, they also detected the crack using an efficient segmentation approach after recognizing the presence of the crack through CNN. Golding et al. [13] evaluated the VGG16 CNN model with different image-processing techniques to identify cracks in concrete structures. The outcomes of this research proved that crack detection did not rely on grayscale or color images. Their results also showed that edge detection and thresholding techniques with CNN reduced the crack recognition ability compared to color images. The best accuracy of this model was 99.54% for color images. To monitor cracks within the building surface structure, Zadeh et al. [14] evaluated various fine-tuned CNN models, including VGG19, ResNet50, InceptionV3, and EfficientNetV2. Their findings demonstrated an impressive accuracy of 99.6% for EfficientNetV2. Regarding detecting cracks in roads and bridges, Kumar et al. [15] generated a CNN model by modifying the LeNet-5 architecture. They assessed their modified LeNet-5 using three concrete crack detection datasets and compared the outcomes with and without employing the principal component analysis (PCA) method. Furthermore, they identified the region of cracks in images. Xu et al. [16] developed a CNN model that included image resolution, multi-scale feature extraction, and complexity minimization to identify the cracks within concrete bridges. Except for pre-training, their CNN provided an overall accuracy of 96.37% for crack identification. Le et al. [17] proposed a CNN model for crack recognition in concrete structures with an overall accuracy of 97.7%. They also demonstrated an integrated framework by merging their intelligent scheme with unmanned aircraft for real-life crack detection. Li and Zhao [18] developed a modified CNN by changing the AlexNet structure for surface crack discovery within the concrete framework. The overall accuracy of this model was 99.09%. This research also integrated their intelligent scheme through a smartphone application to use it in practice. Table 1 summarizes all the relevant works discussed up to this point based on their utilized methods, outcomes, limitations, and future scope. From the analysis of this table, this research enhanced the proposed method to overcome the existing limitations and gaps.

This research utilizes two datasets, each containing images categorized into two groups: crack and non-crack concrete surfaces. These images are preprocessed and proceed through the feature extraction process. As feature extractors, we examine both deep learning (DL) and handcrafted (HC) techniques. From the analysis, we combine the best DL and HC methods to produce a fused feature vector, which is then assessed by feature optimization approaches to identify the most relevant features. A classifier is trained with these relevant features and can recognize the crack and non-crack status of any new concrete surface image. If an image is recognized as containing a crack, our proposed novel algorithm identifies the percentage of the crack within the image and isolates the crack region from other portions of the image. Additionally, two explainable AI (XAI) mechanisms are employed to elucidate the models’ predictions. The key contributions of this research are as follows:

- **Crack recognition:** This research utilizes the fusion of features from two techniques: convolutional neural network (CNNs) and curvelet transform, with a feature optimization mechanism to detect the presence of cracks within an image.
- **Crack region segregation:** Using image processing operations with the convex hull method, we propose a novel algorithm to separate the crack region and identify the crack percentage from an image.
- **Outcome explanation:** We explain the classified images using two popular XAI mechanisms, namely local interpretable model-agnostic explanations (LIMEs) and gradient-weighted class activation mapping++ (Grad-CAM++).

The subsequent sections of this paper are organized as follows: Section 2 details the Materials and Methods used in this research, Section 3 presents the Results and Analysis, and, finally, Section 4 concludes this research.

## 2. Materials and Methodology

**Figure 1:**
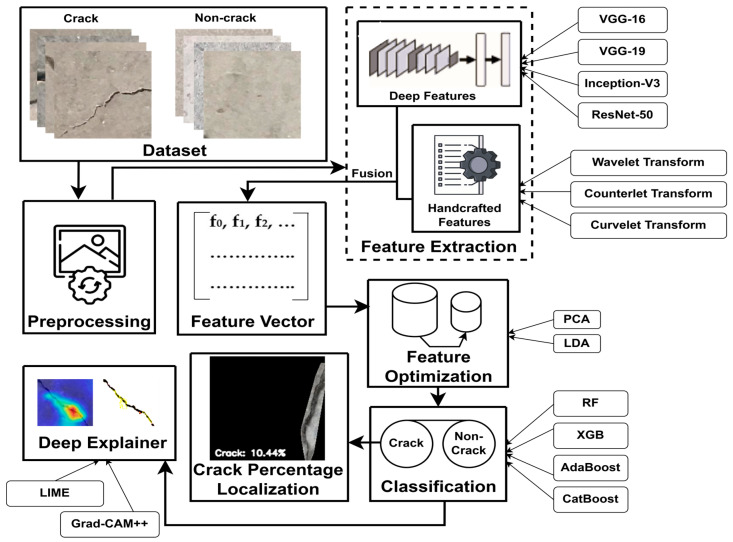
The workflow of this research.

Figure 1 illustrates the research workflow, and Section 2.1 and Section 2.2 elaborate on this research workflow in detail.

### 2.1. Materials

This research utilizes two datasets for all experiments. The first dataset is the surface crack dataset. This dataset is taken from [19]. This dataset contains images of concrete surfaces, categorized into two types: concrete crack surfaces and non-crack surfaces (i.e., normal concrete surfaces). There are a total of 40,000 images in the dataset, each with a size of 227 × 227 × 3 pixels. Of these, 20,000 images depict cracks, while the remaining 20,000 show non-crack surfaces. Therefore, the dataset is balanced. Table 2 presents samples from this dataset. The second dataset is the bridge crack dataset. The source of this dataset is [20]. This dataset contains images of concrete surfaces from bridges. It includes a total of 6069 images, with 4058 crack images and 2011 non-crack images. The images have a size of 224 × 224 × 3 pixels. Table 2 shows sample images from this dataset.

### 2.2. Methods

In the preprocessing stage, we resize all images to 224 × 224 × 3 to meet the requirements of the CNN model. We also use this size for the HC feature extraction method to maintain consistency. To improve feature extraction, we apply a median filter to smooth the images. The median filter effectively removes noise while preserving edges, which is necessary for feature extraction [21].

**Figure 2:**
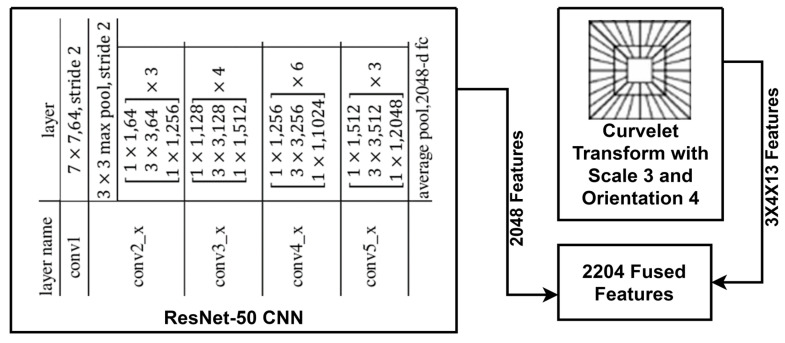
Architecture of the feature extraction technique of this research.

Feature extraction uses techniques to detect and isolate attributes in an image, like edges, textures, shapes, and colors. These features represent the image in a more compact and informative way, helping with image recognition. This research examines both automated (deep learning) and manual (handcrafted) feature extraction methods [22] and fuses these features to identify crack and non-crack images. Figure 2 presents the architecture of the feature extraction process at a glance.

**Figure 3:**
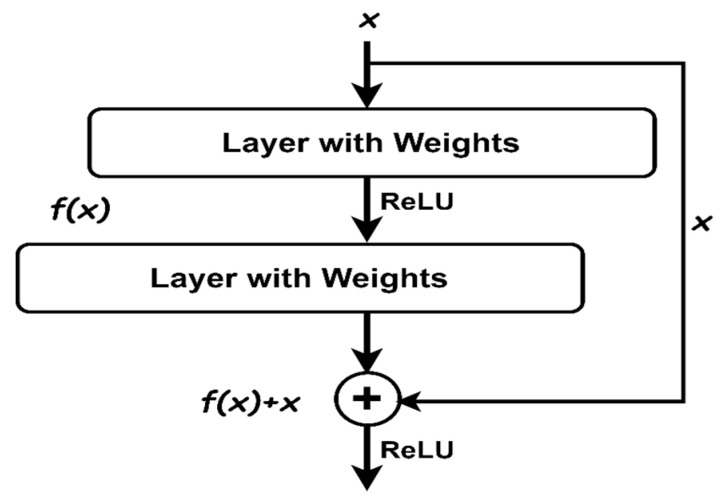
A residual block contains a connection that bypasses two layers.

To extract deep features, this research analyzes several CNN models: VGG-16, VGG-19, Inception-V3, and ResNet-50. CNN models automatically extract significant characteristics from images. For both of our datasets, ResNet-50 performs better than the other CNN models in recognizing concrete crack and non-crack surfaces. ResNet-50 is a deep CNN with 50 layers and is part of the ResNet family introduced by [23]. It addresses the challenge of training deep networks by using residual learning. This method employs shortcut connections to bypass one or more layers, thus mitigating the vanishing gradient problem. These connections are called residual blocks. Figure 3 shows the structure of a residual block. The architecture of ResNet-50 includes an initial 7 × 7 convolutional layer, followed by four stages of residual blocks. Each block contains three layers of 1 × 1, 3 × 3, and 1 × 1 convolutions. These stages gradually increase the number of filters, from 64 to 2048. They include batch normalization and ReLU activation functions. The network ends with a global average pooling layer and a fully connected layer, producing output through a softmax function. Since this research uses ResNet-50 as a feature extractor, we consider the output from the layer just before the final fully connected layer to obtain features. Specifically, this is the output of the global average pooling layer, which follows the fourth stage of residual blocks. This global average pooling layer condenses the spatial dimensions of the feature maps into a single 2048-dimensional feature vector for each input image.

The manual feature extraction approach is essentially the HC method. To extract HC features, this research analyzes three techniques: wavelet transform [24], counterlet transform [25], and curvelet transform [26]. Each technique is applied to the concrete surface images, and then features are extracted from the transformed images using a gray-level co-occurrence matrix (GLCM) [27]. For both of our datasets, the curvelet transform performs better than the other HC methods in recognizing concrete crack and non-crack surfaces. Curvelets represent multi-scale image geometric transformation. Curvelets are preferable over other similar techniques since curvelets represent edges, curves, and directionality effectively. This research employs the wrapping-based fast discrete curvelet transform method, as it is the most efficient approach. For an image *f*[*x*, *y*] with height *M* and width *N*, if *φ*[*x*, *y*] is the curvelet function and *K*1 and *K*2 are the spatial locations of curvelets, then the general expression for the collection of curvelet coefficients is:

(1) $$C\left(j,\theta ,\;k1,\;k2\right)=\sum_{0\leq n<M}^{0\leq y<N}f[x,y]\;\varphi_{j_{,}\theta_{,}{k1}_{,}k2}\left[x,y\right]$$

Here, *j* is the scale and θ is the orientation. For image *f*[*x*, *y*], there exists *j x θ* number of sub-band images [28]. In our method, we used three scale curvelet transforms with four orientations: 0°, 45°, 90°, and 135°. This means that we have a total of (4 × 3) = 12 sub-band images. GLCM is applied to each of these sub-bands. Then, 13 different features, like contrast, correlation, entropy, etc. [27], are calculated from the GLCM-applied sub-band images.

Based on the outcomes, we select the best CNN and HC techniques to form the fused model. We merge features from ResNet-50 and the curvelet transform to create the final feature vector. This results in a total of 2204 features for any concrete surface image. Of these, 2048 features come from the ResNet-50 model, and the rest come from the curvelet transform.

Feature optimization enhances model performance by reducing feature redundancy and noise, thus improving accuracy and decreasing computational complexity. This process ensures the model focuses on the most relevant and informative features, leading to better predictions and efficiency. We use two popular feature optimization techniques, PCA and linear discriminant analysis (LDA) [29], on the fused features. Both techniques enhance performance, with LDA providing the most efficient outcome. LDA reduces dimensionality by finding a new axis that maximizes the separation between different classes. It projects the data onto a lower-dimensional space while maintaining class separability. LDA computes the mean vectors for each class and the overall mean, then calculates the within-class and between-class scatter matrices. It solves an eigenvalue problem to find the linear combinations of features that best separate the classes. The resulting components are ordered by their ability to discriminate between classes, and the top components are used to reduce the dimensionality.

From the final optimized features of a concrete surface image, the eXtreme gradient boosting (XGB) classifier is used to recognize the crack and non-crack status. This research selects XGB due to its superior outcomes after analyzing four different classifiers: XGB [30], random forest (RF) [31], adaptive boosting (AdaBoost) [32], and category boosting (CatBoost) [33]. The XGB classifier builds an ensemble of decision trees sequentially. Each new tree tries to correct the errors made by the previous trees. It uses a technique called gradient boosting. The algorithm calculates the gradient, which is the difference between the predicted and actual values. The new tree is then trained to minimize this gradient. XGB also uses regularization to prevent overfitting. This means it penalizes more complex models to keep them simple. Additionally, it includes techniques like tree pruning and handling missing values. These features make XGB efficient and accurate in classification tasks.

To make the classification outcome understandable, this research uses two deep explainers: LIME and Grad-CAM++. LIME explains predictions by approximating the model locally with a simpler, interpretable model. When using LIME, it perturbs the input data and observes the changes in the output. It then builds a linear model around the prediction to explain it. LIME provides insights into which features are most important for a specific prediction [34]. Grad-CAM++ generates heatmaps to show which parts of an image influence the model’s decision. It calculates the gradients of the target class concerning the feature maps. It then combines these gradients to produce a weighted map. This map highlights the important regions in the image for the prediction. Grad-CAM++ improves on Grad-CAM by better handling multiple instances of the target object in an image. It provides more precise and detailed visual explanations [35]. LIME and Grad-CAM++ help to interpret and visualize how decisions are made in concrete surface crack recognition.

To localize and identify the exact crack region, this research develops an algorithm, which is presented in Algorithm 1. The algorithm begins by converting the input grayscale image *I* to a binary image *B* using a specified threshold *T*. Morphological operations are applied to *B* to enhance crack regions. Contours are then detected in the processed binary image *M*. For each contour, its convex hull *H_i_* is computed. A mask *H* is generated to isolate crack regions by combining all convex hulls. This mask *H* is used to extract crack regions *R* from the original image *I*. To compute the convex hull from contours, Graham’s scan [36] method is used. Graham’s scan is the most popular technique for convex hulls. It begins by selecting the point with the lowest y-coordinate (and the leftmost if tied) as the starting point. It then sorts all other points based on their polar angle relative to this point. Using a stack, it iteratively adds points to form the convex hull, ensuring that each new point does not create a clockwise turn with the last two points on the stack until all points are processed. In Algorithm 1, the percentage of the image area covered by cracks (*P_crack_*) is calculated by comparing the area of *H* to the total area of *I*. Finally, *P_crack_* is returned as the output, providing a quantitative measure of crack presence in the image.
**Algorithm 1.** Crack region isolation using convex hulls**Input:**
Image *I*: A grayscale image representing the input image containing cracks.
Threshold Value *T*: A value used to convert the grayscale image to a binary image.
Minimum Area *A_min_*: Minimum area threshold for considering a contour as a crack region.**Output:**
Image *I*: A grayscale image of the input image containing cracks region with percentage.**Start**

**1.****Convert Image to Grayscale:** Let *G* represent the grayscale image obtained from *I*.
**2.****Apply Binary Thresholding:** Define a binary image B where each pixel is set to-

$B(x,y)=\left\{\begin{array}{l} 1,\;\;if\;G(x,y)\geq T\\ 0,\;\;otherwise \end{array}\right.$
**3.****Enhance Cracks with Morphological Operations:** Use morphological operations on *B* to refine the crack regions. Perform closing and opening operations to smooth and fill gaps in the crack regions.
**4.****Detect Contours:** Identify contours {*C*_1_*, C*_2_, …, *Cn*} in the refined binary image *M*.
**5.****Calculate Convex Hulls:** For each contour *C_i_*, calculate its convex hull *Hi*

$H_{i}=\mathrm{ConvexHull}\left(C_{i}\right)$
**6.****Create Convex Hull Mask:** Create a mask *H* where each pixel belongs to one or more convex hulls.

$H(x,y)=\left\{\begin{array}{l} 1,\;\;if(x,y)\;is\;inside\;any\;convex\;hull\;H_{i}\\ 0,\;\;otherwise \end{array}\right.$
**7.****Isolate Crack Regions**: Generate an image *R* by masking *I* with *H.* So, *R* now contains only the crack regions isolated from the original image *I*.

R(x,y)=I(x,y)⋅H(x,y)
**8.****Calculate Crack Percentage:** Determine the percentage of the image area covered by cracks-

$P_{\mathrm{crack}\;}=\frac{\;\mathrm{Total}\;\mathrm{number}\;\mathrm{of}\;\mathrm{pixels}\;\mathrm{set}\;\mathrm{to}\;1\;\mathrm{in}\;\mathrm{mask}\;H.}{\;\mathrm{Total}\;\mathrm{number}\;\mathrm{of}\;\mathrm{pixels}\;\mathrm{in}\;\mathrm{the}\;\mathrm{original}\;\mathrm{image}\;I}\times 100$
**9.****Output:** Return P*_crack_*, representing the percentage of the image area covered by cracks.**End**


## 3. Experimental Results and Analysis

This section presents the outcome of this research in detail.

### 3.1. Criteria for Assessing Performance

To evaluate the performance of the proposed method, we employ 10-fold cross-validation [37] with a training-to-testing ratio of 8:2 on the datasets. The performance metrics used include accuracy, precision, recall, specificity, F1-score, normalized confusion matrix (NCM), and ROC curve [38]. These metrics are detailed below.

- Accuracy measures the proportion of correctly classified instances.
  (2) $$Accuracy\;=\frac{TP+TN}{TP+TN+FP+FN}$$
  where TP is the number of crack images correctly identified as cracks, TN is the number of non-crack images correctly identified as non-cracks, FP is the number of non-crack images incorrectly identified as cracks, and FN is the number of crack images incorrectly identified as non-cracks from all the testing images.
- Precision indicates the proportion of true-positive results among all positive predictions.
  (3) $$Precision\;=\frac{TP}{TP+FP}$$
- Recall measures the ability to correctly identify positive instances.
  (4) $$Recall\;=\frac{TP}{TP+FN}$$
- Specificity assesses the ability to correctly identify negative instances.
  (5) $$Specificity\;=\frac{TN}{TN+FP}$$
- F1-score is the harmonic means of precision and recall.
  (6) $$F1\text{-}score\;=2\times \frac{Precision\times Recall}{Precision+Recall}$$
- NCM: Tabular form presents the percentage of TP, TN, FP, and FN.
- ROC curve plots the true-positive rate (recall) against the false-positive rate (1-specificity) at various threshold settings, helping to visualize the trade-off between sensitivity and specificity.

### 3.2. Outcome of Crack Recognition

Table 3 presents the performance of various techniques for the surface crack dataset. The analysis of Table 3 indicates that the feature extractor ResNet-50 achieves the highest accuracy of 99.38%, surpassing all other individual feature extraction methods. Additionally, HC feature extractors such as wavelet transform, counterlet transform, and curvelet transform perform worse than CNN techniques. Among HC methods, the curvelet transform achieves a peak accuracy of 87.24%, whereas VGG-19, the worst-performing CNN mechanism, achieves 98.36% accuracy. The fusion of the best CNN and HC methods is the combination of ResNet-50 and curvelet transform, which results in an accuracy of 99.63%. With feature optimization using PCA and LDA, this fused mechanism achieves 99.76% and 99.93% accuracy, respectively. Therefore, the fusion of ResNet-50 and curvelet transform with LDA is the ultimate crack recognition approach using the surface crack dataset.

Table 4 delineates the performance metrics of various techniques applied to the bridge crack dataset. Analyzing Table 4 reveals that the ResNet-50 feature extractor attains an apex accuracy of 99.01%, thus outperforming all other standalone feature extraction techniques. In contrast, HC feature extractors, encompassing the wavelet transform, counterlet transform, and curvelet transform, exhibit inferior performance relative to CNN-based techniques. Among HC methods, the curvelet transform registers a maximum accuracy of 89.78%, whereas VGG-16, the least efficacious CNN model, accomplishes an accuracy of 97.53%. The amalgamation of the preeminent CNN and HC methods, that is, the combination of ResNet-50 and curvelet transform, culminates in an accuracy of 99.09%. Subsequent to feature optimization via PCA and LDA, this fused mechanism realizes accuracies of 99.17% and 99.69%, respectively. Consequently, the integration of ResNet-50 and curvelet transform with LDA emerges as the paramount crack recognition strategy for the bridge crack dataset.

**Figure 4:**
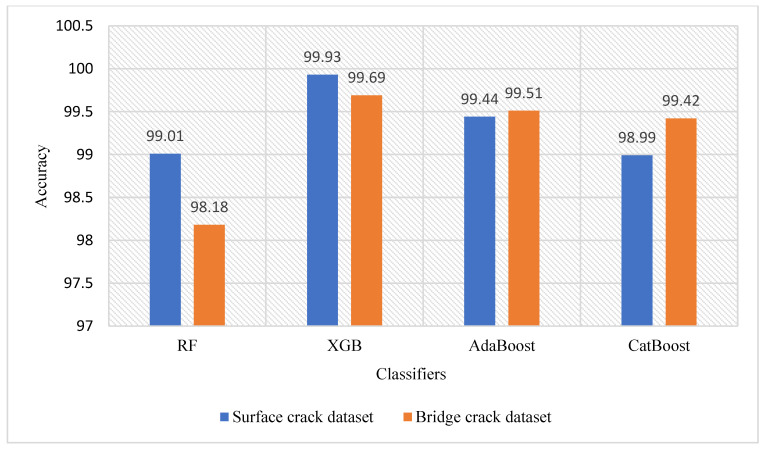
Performance analysis of different classifiers.

This research employs the XGB classifier due to its superior performance compared to other methods, namely RF, AdaBoost, and CatBoost. Figure 4 presents an accuracy comparison of different classifiers using the proposed method (ResNet-50 and curvelet transform with LDA) across both datasets. The analysis of Figure 4 indicates that XGB achieves the highest accuracy, with 99.93% and 99.69% for the surface crack dataset and bridge crack dataset, respectively. AdaBoost holds the second highest results, with 99.44% and 99.51% for the surface crack dataset and bridge crack dataset, respectively. The CatBoost classifier records the third highest accuracy, with 98.99% and 99.42% for the surface crack dataset and bridge crack dataset, respectively. However, the RF classifier yields the poorest performance among the classifiers, with overall accuracies of 99.01% and 98.18% for the surface crack dataset and bridge crack dataset, respectively.

This research uses 10-fold cross-validation to assess the proposed method. This technique is vital for evaluating a model’s robustness and generalizability by dividing the dataset into 10 subsets. The model is trained on nine of these subsets and tested on the remaining one, with this process repeated for each subset. This approach helps to minimize overfitting and provides a more accurate performance measure, as every data point is used for both training and validation. Table 5 and Table 6 show the performance of the proposed method using 10-fold cross-validation for the surface crack dataset and bridge crack dataset, respectively. The analysis of these tables demonstrates that the proposed method exhibits minimal variation and maintains relatively consistent performance.

**Figure 5:**
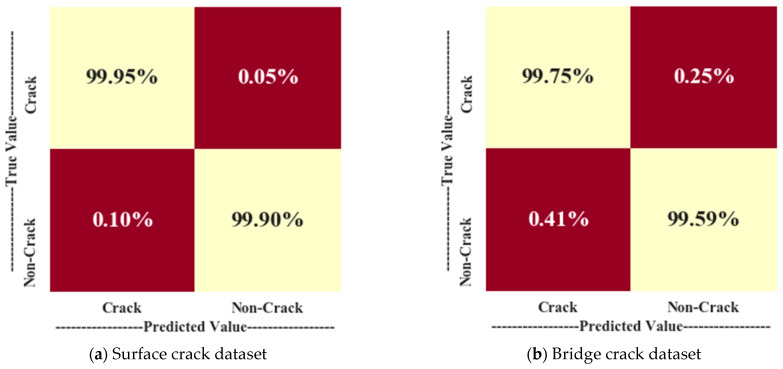
Normalized confusion matrix of the proposed method.

Figure 5 displays the NCM of the proposed method for both datasets. Figure 5a shows that 99.95% of crack images and 99.90% of non-crack images are correctly identified in the surface crack dataset during testing. Figure 5b indicates that in the bridge crack dataset, 99.75% of crack images and 99.59% of non-crack images are correctly identified during testing. The analysis of Figure 5 also reveals that 0.05% of crack images and 0.10% of non-crack images are misclassified in the surface crack dataset, while 0.25% of crack images and 0.41% of non-crack images are misclassified in the bridge crack dataset.

**Figure 6:**
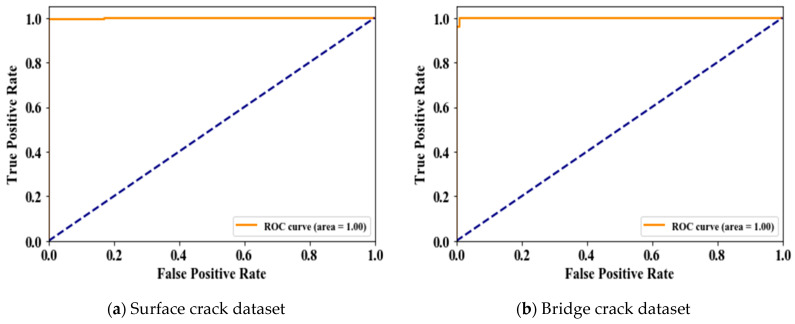
ROC curve of the proposed method.

Figure 6 illustrates the ROC curves for the proposed method applied to two datasets: surface cracks (Figure 6a) and bridge cracks (Figure 6b). The ROC curve is a graphical representation that plots the true-positive rate (TPR) against the false-positive rate (FPR) at various classification thresholds, assessing the model’s ability to distinguish between crack and non-crack images. The diagonal blue line represents a random classifier, which serves as a baseline. In both subfigures, the orange curve is perfectly aligned with the top-left corner of the plot, indicating that the proposed method achieves an AUC (Area Under the Curve) of 1.0 for both datasets. An AUC of 1.0 signifies that the model performs flawlessly, with perfect sensitivity (no false negatives) and specificity (no false positives). This result demonstrates that the proposed method is highly effective and reliable in correctly identifying cracks in both the surface and bridge datasets without any errors, reflecting its robust performance in binary classification tasks.

### 3.3. Outcome of Crack-Region Segregation

After identifying a crack image using the ResNet-50, curvelet transform, and LDA-based approach, we determine the percentage of the crack in that image using Algorithm 1. Table 7 displays the results of Algorithm 1 for some sample images from both datasets. The outputs in Table 7 demonstrate that our convex hull-driven algorithm not only identifies the percentage of the crack but also isolates the crack region from the other portions of a crack image.

### 3.4. Outcome Explanation

Table 8 explains the crack images using Grad-CAM++ and LIME for the deep model employed in the proposed method. The outcome of Grad-CAM++ in this table generates more precise and visually detailed heatmaps by considering higher-order derivatives of the loss concerning the feature maps. This makes it particularly effective in highlighting important regions in images where crack features are present. The explanation of LIME in Table 8 highlights regions of the crack image that are most influential in the model’s decision-making process, and this is nothing but the exact crack region.

### 3.5. Comparative Analysis

**Figure 7:**
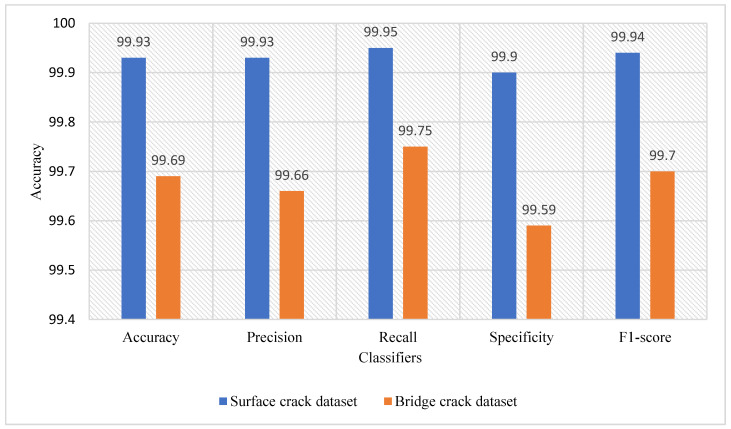
Comparison of the performance of the proposed method in surface crack dataset and bridge crack dataset.

The primary objective of this research is to identify the presence of cracks, along with the damage percentage and area, in any concrete structure. We utilize two datasets of concrete structures containing both cracked and normal images. The surface crack dataset pertains to building structures, while the bridge crack dataset pertains to bridge structures. Based on an analysis of several recent techniques, we develop our final model to recognize cracks in images through the fusion of ResNet-50 CNN and curvelet transform-based feature extraction, combined with LDA and XGB classifiers. Our proposed approach effectively identifies cracks in both datasets, performing particularly well with the surface crack dataset. Figure 7 shows a performance comparison of the proposed method in both datasets. Once a crack is identified, the proposed algorithm employing convex hull thresholding, morphological operations, and contour finding mechanisms plots the percentage of the crack in the image. Additionally, this research experiments with various XAI methods to explain the crack recognition process.

Table 9 compares this research with other SOTA methods based on various criteria, such as results, ability to segregate the crack region, crack percentage localization, and outcome explanation capability. The analysis of this table shows that existing methods are mostly developed based on a single dataset, whereas this work uses two different datasets. Decisions based on a single dataset are not sufficient to draw any vital conclusions; hence, the use of two datasets by the proposed method, along with a proper outcome assessment, is a significant contribution.

Table 9 also indicates that the methods by Akgül [11], Padsumbiya et al. [12], Golding et al. [13], Zadeh et al. [14], and Le et al. [17] all use the surface crack dataset, with the best accuracy provided by Akgül [11] at 99.87%. The accuracy of our method for the surface crack dataset is 99.93%, which is better than Akgül’s [11]. For the bridge crack dataset, the best accuracy of 99% is presented by Kumar et al. [15], which is lower than the proposed method’s accuracy of 99.69%. Another method by Li and Zhao [18] uses a private dataset, achieving an accuracy of 99.06%, which is still less than the accuracy of our method. Therefore, in terms of accuracy, the proposed method outperforms all techniques, highlighting another significant contribution.

The analysis in Table 9 shows that while the methods by Padsumbiya et al. [12] and Kumar et al. [15] can segregate the crack region within the image, none of the SOTA methods can detect the percentage of the crack in the image. We developed an algorithm for this purpose. Moreover, none of the techniques in Table 9, except for this research, can explain the outcome through XAI. This research is the first to use the XAI technique with an image-based intelligent crack recognition technique. Therefore, in terms of crack percentage visualization and outcome explanation, the proposed research shows significant contributions.

In addition, some efficient methods that are not fully aligned with this research also offer significant improvements, but the proposed work outperforms these techniques too. For instance, Shang et al. [39] used support vector machine (SVM) and fused CNN Long Short-Term Memory (LSTM) models in signal and image data to detect localized damage in plate structures. They generated the image dataset by converting signal data. Although both SVM and CNN-LSTM achieved 100% accuracy, their work lacks cross-validation and does not provide an explanation for the outcomes. Wu et al. [40] presented an enhanced CNN architecture called MobileNetV2_DeepLabV3 to measure dam crack width from images through segmentation. The model achieves an intersection rate of 83.23%. However, the research does not provide the percentage of the crack or an explanation of the segmentation outcome. In their paper, Wan et al. [41] present various methods for monitoring bridge health. The analysis of these methods reveals a lack of explanatory capability. Ozturk [42] investigates the seismic behavior of two historic temple buildings in the Cappadocia region of Turkey, using dynamic analysis based on ground motion records from recent Turkish earthquakes. The analysis considers the impact of structural walls and highlights that slab discontinuities on the first floors are a major factor in expected structural damage. Significant deformation is observed in the roof domes of the Konakli building, with destructive levels of drift contributing to the anticipated damage. The study cannot ensures any intelligence related to damage recognition. Besides, several other efficient methods [43,44,45,46,47,48,49,50] also provide proficient techniques to identify cracks from images, but none of them is capable of explaining the outcome and capturing the crack percentage.

## 4. Conclusions

This study presents a novel approach for concrete surface crack detection by integrating deep learning and handcrafted features with a unique convex hull-driven technique for crack percentage isolation. The fusion of ResNet-50 CNN-derived deep features with handcrafted curvelet transform features, followed by optimization using PCA and LDA, and classification through XGBoost, demonstrated superior performance. The model achieved a remarkable accuracy of 99.93% and 99.69% on two distinct datasets, showcasing the effectiveness of the proposed method. Additionally, the incorporation of explainability techniques like LIME and Grad-CAM++ provided deeper insights into the decision-making process of the model, making the predictions more transparent and interpretable. The practical application of this research lies in the automated and accurate detection of cracks in concrete surfaces, which is critical for maintaining structural integrity in civil engineering. The ability to isolate and quantify the crack percentage offers engineers a powerful tool for assessing the extent of damage, thereby enabling more informed decisions regarding maintenance and repairs. This approach not only reduces the need for manual inspections but also minimizes the subjectivity and inconsistency associated with traditional methods, leading to more reliable outcomes in real-world scenarios. For civil engineering professionals, it is recommended to adopt this AI-based crack detection framework as part of regular structural assessments. The method can be integrated into existing infrastructure monitoring systems to enhance the accuracy and reliability of crack detection processes. Additionally, the explainability aspect of the model should be leveraged to ensure that engineers understand the basis of AI predictions, facilitating trust and broader acceptance of AI-driven tools in structural diagnostics. In our future work, we plan to integrate our method with real-time monitoring systems, extending its applicability to diverse structural types and materials. In addition to identifying crack percentages, we will also try to quantify additional parameters of cracks, such as their width and length.

## Figures and Tables

**Table 1:** Summary of relevant prior research.

Method	Technique	Accuracy	Limitation	Future Scope
Akgül [11]	MobileNetV2 and DenseNet169 based novel CNN	99.87%	No fold cross-validation and outcome explanation	Employing for images having noise or obstacles
Padsumbiya et al. [12]	Four layers sequential CNN model	97.8%	Lack of comparative outcome analysis and outcome explanation	Employing to multiple surface distresses
Golding et al. [13]	VGG16 CNN model	99.54%	No outcome interpretation and cross-validation. Limited to a particular CNN model only	Evaluating thresholds of 106 and 101 to images and employing VGC11, VGC19, and AlexNet CNN architectures
Zadeh et al. [14]	Fine-tuned EfficientNetV2 CNN model	99.6%	Single-fold data evaluation and lack of outcome explanation	--
Kumar et al. [15]	Modified LeNet-5 CNN model	99%	Inexplicable the causes of crack occurrence and no cross-validation for outcome	Enhancing the model to elucidate the underlying causes of crack occurrence
Xu et al. [16]	Lower complex CNN model with high-resolution image managing and multi-scale feature extraction modules	96.37%	Incapability to draw crack region; lack of outcome evaluation and explanation	Deploying the proposed CNN to other convolutional networks as a feature extraction module
Le et al. [17]	CNN	97.7%	Incapability of outcome reasoning and cross-validation as well as lack of model novelty	Employing for multiple types of cracks; Exploring diverse advanced models
Li and Zhao [18]	Modified AlexNet	99.06%	No outcome interpretation and single-fold data evaluation	Exploring the model to analyze a broader range of concrete defects across diverse environmental conditions

**Table 2:** Sample images from the datasets.

Surface Crack Dataset		Bridge Crack Dataset	
Crack	Non-Crack	Crack	Non-Crack
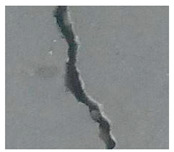	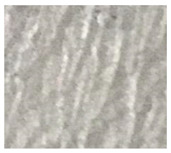	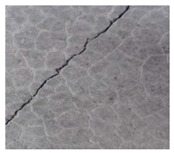	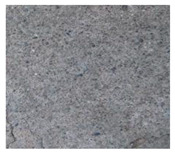
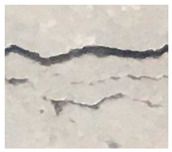	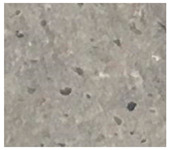	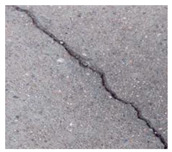	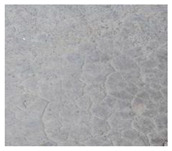
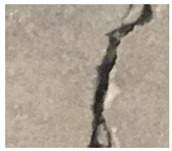	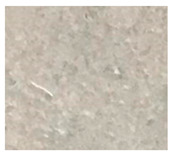	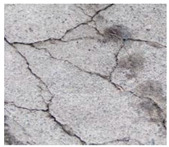	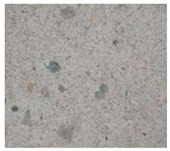

**Table 3:** Performance of different techniques for surface crack dataset.

Technique	Accuracy	Precision	Recall	Specificity	F1-Score
VGG-16	98.50	99.14	97.57	99.28	98.35
VGG-19	98.36	98.35	98.06	98.61	98.20
Inception-V3	99.05	98.82	99.09	99.01	98.96
ResNet-50	99.38	99.21	99.45	99.33	99.32
Wavelet transform	79.21	78.29	80.14	78.30	79.20
Counterlet transform	83.78	82.75	85.57	81.98	84.14
Curvelet transform	87.24	87.25	87.91	86.53	87.57
ResNet-50 + Curvelet transform	99.63	99.67	99.53	99.72	99.60
ResNet-50 + Curvelet transform + PCA	99.76	99.70	99.78	99.74	99.74
ResNet-50 + Curvelet transform + LDA	99.93	99.93	99.95	99.90	99.94

**Table 4:** Performance of different techniques in bridge crack dataset.

Technique	Accuracy	Precision	Recall	Specificity	F1-Score
VGG-16	97.53	98.16	96.86	98.19	97.50
VGG-19	98.43	97.70	99.16	97.71	98.43
Inception-V3	98.92	99.50	98.36	99.50	98.93
ResNet-50	99.01	98.51	99.50	98.52	99.00
Wavelet transform	84.51	87.80	81.36	87.88	84.46
Counterlet transform	87.39	90.39	85.17	89.87	87.71
Curvelet transform	89.78	92.98	87.55	92.36	90.18
ResNet-50 + Curvelet transform	99.09	99.33	98.84	99.34	99.09
ResNet-50 + Curvelet transform + PCA	99.17	99.50	98.84	99.51	99.17
ResNet-50 + Curvelet transform + LDA	99.69	99.66	99.75	99.59	99.70

**Table 5:** Performance of the proposed method evaluated on a fold-by-fold basis for the surface crack dataset.

Fold	Accuracy	Precision	Recall	Specificity	F1-Score
fold-1	99.9	99.89	99.93	99.84	99.91
fold-2	99.98	99.98	100	99.94	99.99
fold-3	99.87	99.89	99.84	99.9	99.86
fold-4	99.96	99.95	99.97	99.93	99.96
fold-5	99.96	99.96	99.98	99.93	99.97
fold-6	99.94	99.93	99.95	99.91	99.94
fold-7	99.91	99.88	99.95	99.86	99.91
fold-8	99.95	99.95	99.95	99.94	99.95
fold-9	99.97	99.98	99.97	99.97	99.97
fold-10	99.88	99.9	99.95	99.73	99.92
Average	99.93	99.93	99.95	99.90	99.94

**Table 6:** Performance of the proposed method evaluated on a fold-by-fold basis for bridge crack dataset.

Fold	Accuracy	Precision	Recall	Specificity	F1-Score
fold-1	99.91	99.85	100	99.81	99.92
fold-2	99.83	99.86	99.86	99.78	99.86
fold-3	99.75	99.44	99.71	99.76	99.58
fold-4	99.58	99.53	99.88	98.89	99.7
fold-5	99.51	99.86	99.31	99.79	99.58
fold-6	99.75	99.16	100	99.65	99.58
fold-7	99.42	99.84	99.08	99.82	99.46
fold-8	99.83	99.73	100	99.56	99.86
fold-9	99.67	99.61	99.87	99.31	99.74
fold-10	99.67	99.74	99.74	99.54	99.74
Average	99.69	99.66	99.75	99.59	99.70

**Table 7:** Illustration of various stages of crack percentage detection.

Sample Source	Actual Image	Binary Image	Morphological Image	Isolated Crack Regions
Surface crack dataset	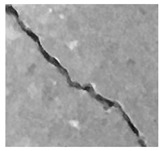	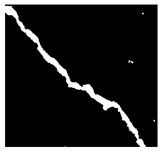	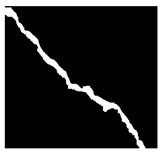	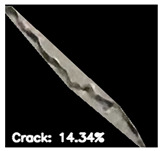
Surface crack dataset	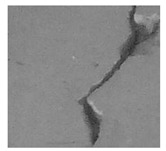	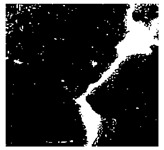	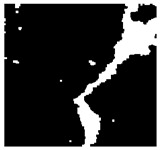	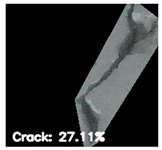
Bridge crack dataset	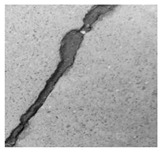	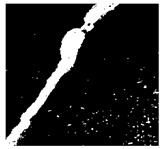	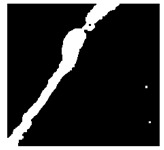	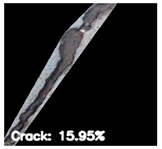
Bridge crack dataset	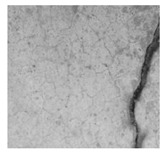	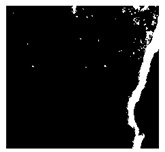	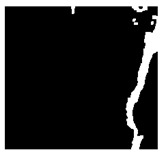	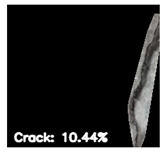

**Table 8:** Explanation of the crack images using XAI techniques.

Sample Source	Actual Image	Grad-CAM++	LIME
Surface crack dataset	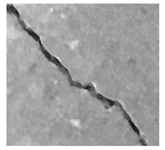	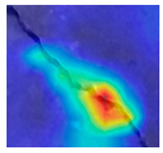	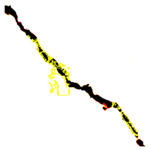
Surface crack dataset	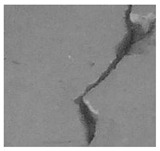	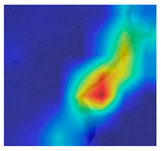	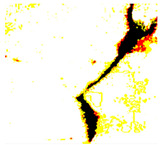
Bridge crack dataset	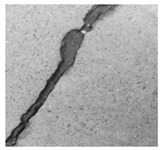	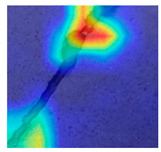	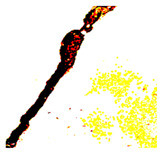
Bridge crack dataset	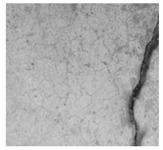	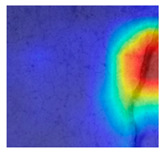	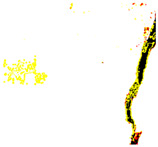

**Table 9:** Comparison of various methods.

Method	Dataset	Accuracy	Crack Percentage Detection	Crack Region Segregation	Utilization of XAI
Akgül [11]	Surface crack dataset	99.87%	X	X	X
Padsumbiya et al. [12]	Surface crack dataset	97.8%	X	✓	X
Golding et al. [13]	Surface crack dataset	99.54%	X	X	X
Zadeh et al. [14]	Surface crack dataset	99.6%	X	X	X
Kumar et al. [15]	Bridge crack dataset	99%	X	✓	X
Xu et al. [16]	Bridge crack dataset	96.37%	X	X	X
Le et al. [17]	Surface crack dataset	97.7%	X	X	X
Li and Zhao [18]	Private dataset of 1455 images of 4160 × 3120	99.06%	X	X	X
Proposed method	Surface crack dataset	99.93%	✓	✓	✓
	Bridge crack dataset	99.69%			

## Data Availability

The source of the datasets is provided in Section 3.1.
